# Role of C-C chemokine receptor type 7 and its ligands during neuroinflammation

**DOI:** 10.1186/1742-2094-9-77

**Published:** 2012-04-25

**Authors:** Shahani Noor, Emma H Wilson

**Affiliations:** 1 Division of Biomedical Sciences, Center for Glial-Neuronal Interactions, University of California Riverside, 900 University Ave, Riverside, CA, 92521, USA

**Keywords:** CCR7, Infection, Neuroinflammation, T cell trafficking, CCL21

## Abstract

For decades, chemokines and their receptors have received a great deal of attention for their multiple roles in controlling leukocyte functions during inflammation and immunity. The ability of chemokines to convey remarkably versatile but context-specific signals identifies them as powerful modulators of immune responses generated in response to diverse pathogenic or non-infectious insults. A number of recent studies have speculated that the C-C chemokine receptor type 7 (CCR7), plays important roles in immune-cell trafficking in various tissue compartments during inflammation and in immune surveillance. Using computational modeling and microfluidics-based approaches, recent studies have explored leukocyte migration behavior in response to CCR7 ligands in a complex chemokine environment existing with other coexisting chemokine fields. In this review, we summarize the current understanding of the effects of soluble versus immobilized ligands and of the downstream signaling pathways of CCR7 that control leukocyte motility, directionality, and speed. This review also integrates the current knowledge about the role of CCR7 in coordinating immune responses between secondary lymphoid organs and peripheral tissue microenvironments during primary or secondary antigen encounters. CCR7 seems to influence distinct immunological events during inflammatory responses in the central nervous system (CNS) including immune-cell entry and migration, and neuroglial interactions. The clinical and pathological outcome may vary depending on its contribution in the inflamed CNS microenvironment. Understanding these mechanisms has direct implications for therapeutic developments favoring more protective and efficient immune responses.

## Background

CCR7 is a G-protein-coupled receptor that is expressed mainly on semi-mature and mature dendritic cells (DCs), naive B and T cells and central memory T cells [1-5]. The CCR7 ligands, CCL19 and CCL21, are constitutively expressed on the high endothelial vessels (HEVs) of the lymph nodes (LNs) and by fibroblastic reticular cells and follicular dendritic cells forming the conduits that guide T-cell migration in the LN [6,7]. Hence, CCR7 is the principal chemokine receptor that controls DC–T-cell antigen-specific interactions facilitating optimal priming in the LN [2,8-10]. In addition to its role in development and maintenance of secondary lymphoid organs, CCR7 signaling is involved in a number of immunological processes such as the generation of thymocytes [11,12], central and peripheral tolerance [13,14], regulatory T-cell (Treg) function [15-17] and T-cell homeostasis [18] (Table 1).

**Table 1:** Summary of roles of CCR7

**Role in the immune system**	**Mechanism**	**References**
Thymic architecture and function	CCR7 is involved in the recruitment of fetal hematopoietic progenitors and coordination of migratory events of thymocytes at their different maturation and selection ages in the thymus	[11,12,19-21]
Regulatory T-cell function	CCR7 is required for Treg cell homing and positioning within the paracortical LN area. Treg function is impaired in CCR7-deficient mice	[15-17]
T-cell priming	Age-experienced DCs (entering via afferent lymphatics) and T cells (via HEVs) use CCL21-coated stromal networks in the T-cell zone to interact with each other and generate the effector T-cell pool	[6,7,22,23]
Lymphocyte recirculation in peripheral tissues	CCR7 and CCL21 contribute to T-cell recruitment and egress from peripheral tissues, and the pleural and the peritoneal cavities	[24-28]
Peripheral tissue- resident DC trafficking	Tolerogenic (homeostatic) DCs and inflammation-induced DCs acquire CCR7 expression as they exit from peripheral tissues to present antigens in the draining LN	[3,14,29,30]
DC survival, maturation and antigen uptake	CCR7-mediated signaling positively regulates the survival and rate of endocytosis of the mature DCs. CCR7 also induces dendritic cytoplasmic extensions that may contribute to the ability of DCs to present antigens	[31-33]
T-cell homeostasis	CCR7 ligands support the survival and homeostatic expansion of naive T cells	[18,34]
B-cell help	CCR7 upregulation mobilizes follicular B cells towards the T-cell zone in the LN to receive ‘help’ from CD4+ T helper cells	[35,36]

*CCR7* C-C chemokine receptor 7; *DC* dendritic cell; *HEV* high endothelial vessel; *LN* LN.

In recent years, considerable effort has been devoted to understanding the mechanisms of how CCR7 signaling components control leukocyte propulsion and directional migration [37-40]. These mechanisms have identified CCR7 ligands as potent regulators of leukocyte interactions in lymphoid tissues and peripheral tissues including the CNS.

In this review, we focus on these properties of CCR7/CCL21 signaling and its role in generating protective immune responses, particularly those occurring in the CNS.

### Signaling properties of CCR7 and its ligands

CCR7 is a seven trans-membrane domain receptor protein coupled with pertussis toxin-sensitive G_ai_ components. Signaling of immobilized CCR7 ligands through their receptor causes lymphocytes to go from a ‘rolling’ state to an ‘arrest’ state on endothelial surfaces, and subsequent activation and redistribution of integrin molecules leads to lymphocyte transendothelial migration [2,41]. CCR7 signaling components have multiple features that allow them to govern a wide range of leukocyte functions in different tissue microenvironments. An important aspect of the CCR7 chemokine system is its transcriptional regulation. Although CCR7 has been established as a homeostatic chemokine receptor, both CCR7 and its ligands are also inducible during inflammation to coordinate complex leukocyte trafficking between the peripheral versus the lymphoid tissue [24,42,43]. Importantly, ligation of CCL19 versus CCL21 may allow CCR7 to exert differential effects within tissue for the following reasons.

**Figure 1:**
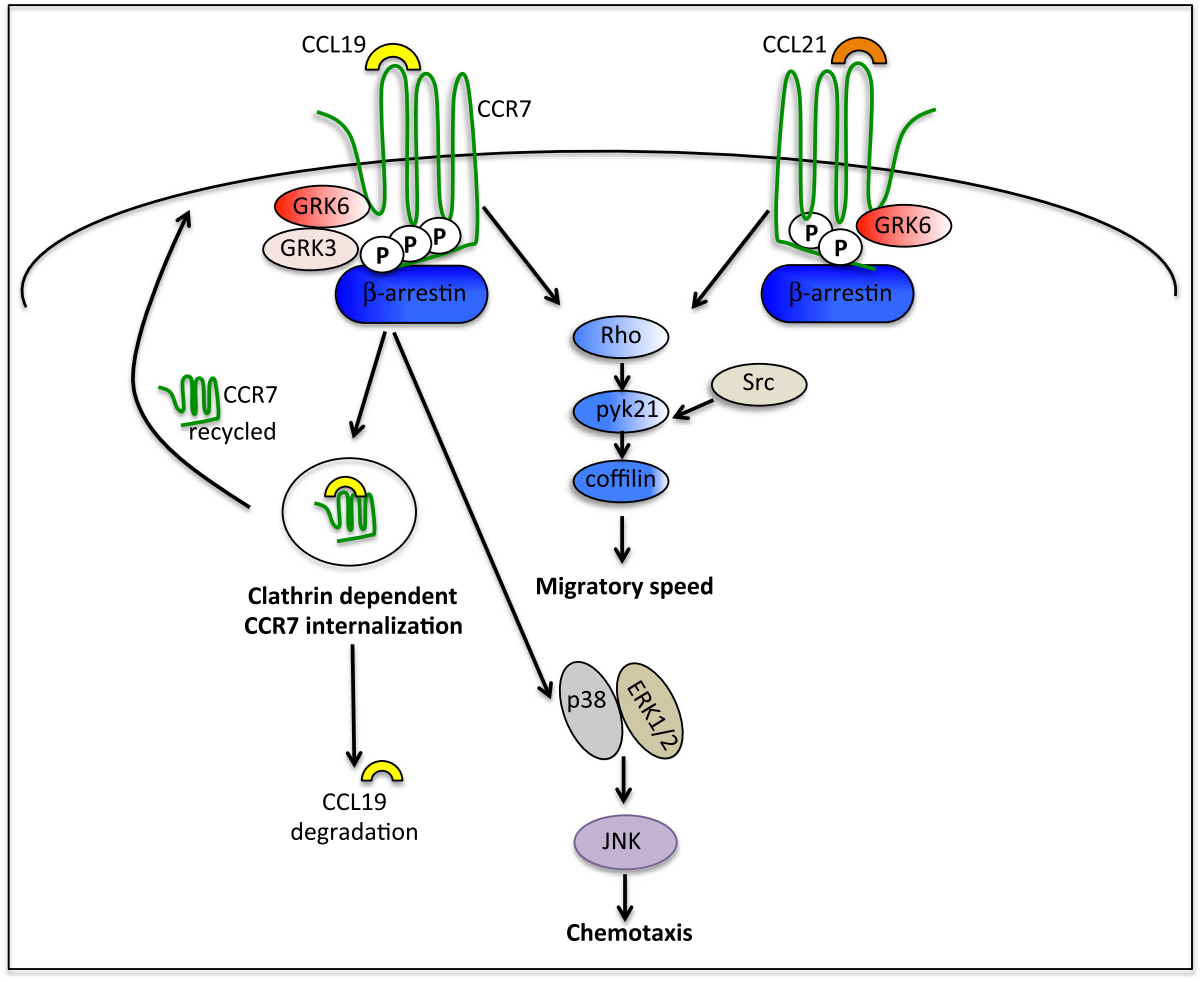
**Differential consequences of CCL19 versus CCL21 ligation to C-C chemokine receptor 7.** CCR7 signaling activates the mitogen-activated protein kinase signaling module leading to chemotaxis, whereas the Rho-coffilin signaling axis is involved in controlling the migratory speed of leukocytes. Ligand binding to and activation of CCR7 leads to its phosphorylation by GRKs that recruit β-arrestin scaffold proteins. Signaling by both CCL19 and CCL21 causes GRK6 to phosphorylate CCR7. In addition, GRK3 phosphorylates CCR7 after CCL19 ligation only. The differential phosphorylation pattern may recruit distinct functional pools of β-arrestins that leads to the differential ability of CCR7 ligands to induce clathrin-dependent receptor endocytosis and desensitization. After internalization, CCR7 recycles back to the plasma membrane, whereas CCL19 is sorted to lysosomes for degradation.

1) CCL21 has an affinity more than 10-fold higher than CCL19 for binding collagen and other extracellular molecules, thus allowing it to be a better candidate to form an immobilized chemokine gradient [44].

2) CCL19 and CCL21 are natural biased ligands of CCR7, which have equivalent efficacy for G-protein activation but possess differential engagement of the G-protein-coupled receptor kinase (GRK)/β-arrestin system [45] and hence presumably differentially phosphorylate CCR7 [46] (Figure 1). Thus, CCL19 but not CCL21 binding to CCR7 induces receptor desensitization and clathrin-mediated internalization [47-49]. This may result in local changes in the chemokine environment that would optimize directed immune responses.

3) Differential expression and localization of CCL19 and CCL21 in different regions of the tissue have a functional significance that can influence the position of lymphocytes and DCs in the LN and spleen tissue microenvironment [25,39].

4) CCL21 and CCL19 are the only ligands for CCR7. However, CCL21 can also signal through CXCR3, a prominent inflammatory chemokine receptor in Th1 immune responses [50,51].

5) CCR7 ligands share only 32% sequence homology. CCL19 is an obligate soluble ligand, whereas CCL21 remains membrane-bound because of the presence of a glycosaminoglycan binding domain [37,52-54]. These soluble and immobilized ligands therefore have the potential to induce different functional responses even though they signal through the same receptor.

CCR7 downstream signaling uses multiple independent modules to control distinct leukocyte functions (Figure 1). In DCs, chemotaxis toward CCR7 ligands involves G-inhibitory-mediated activation of mitogen-activated protein kinase family members, and their migratory speed is controlled by Rho–coffilin phosphorylation [55,56]. Thus, actin and myosin inhibitors affect only the speed of crawling, whereas pertussis toxin inhibits the directed motion of bone-marrow-derived DCs [38].

**Figure 2:**
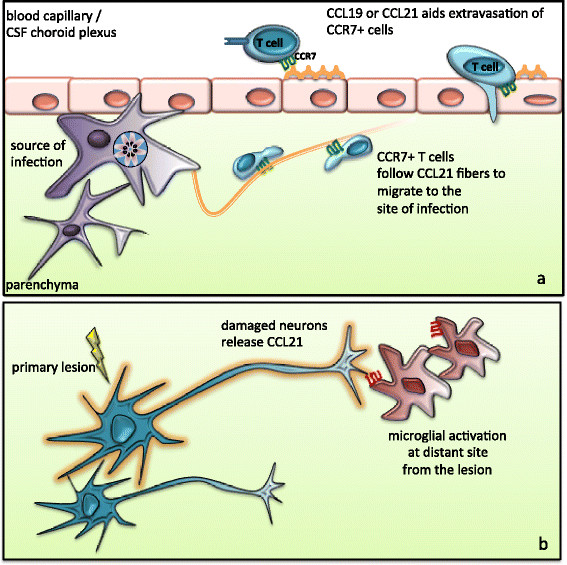
**Models proposing the role of CCL21 during inflammation in the brain. (a)** CCL19 and CCL21 expression at the blood–brain barrier (BBB) may aid extravasation of CCR7+ leukocytes. During pathogenic insults, CNS-resident glial cells induce CCL21 which in turn facilitates T-cell migration from the perivascular area to the site of infection to keep pathogens under control; **(b)** Ischemia- or glutamate-mediated damage causes neurons to release CCL21 from the primary lesion site to activate microglia through the CXCR3 receptor. CXCR3-CCL21 signaling-mediated neuroglial communication is a potent mechanism to activate glial cells that are present at a distant site from the lesion.

Recently, significant conceptual advances have been made, based on *ex vivo* and *in vitro* experimental models investigating how soluble versus immobilized CCR7 ligands influence the speed, motion, and directionality of leukocyte movement within lymphoid tissues [37,39,40]. Studies conducted using two-dimensional microfluidic devices have shown that CCL19 is apparently 100-fold more potent than CCL21 for DC chemotaxis [38], and is also a more potent chemoattractant than CXCL12 for activated T cells [57]. By contrast, another recent study used microfluidics-based approaches to generate chemokine gradients on three-dimensional (3D) matrices mimicking the 3D tissue environment *in vivo*[40]. This latter study found that when CCL21 and CCL19 are presented on competing overlapping gradients, DC migration will follow CCL21. A similar preference is seen in human peripheral blood T cells that are chemotactic to CCL21 but not CCL19 under physiological gradient conditions [39]. The differential ability of CCL19 and CCL21 to desensitize the receptor may fine-tune the intensity of CCR7 signaling in coexisting chemokine fields, potentially explaining the preferential migration to CCL21. Furthermore, DCs preferentially chemotax towards CCL21 even if not bound to the tissue matrix [40]. In support of these data, using a 3D gel carbon-fiber system, Schumann *et al.* found that the adhesive random migration of DCs is mediated through immobilized CCL21, but, the directional cues come from a soluble form of CCL21 generated from protease cleavage by the migrating DCs [37]. Although the authors showed the existence of a truncated form of CCL21*,* the question remains whether this soluble form is produced homogenously or as a gradient *in vivo*, and if the latter, how this gradient is formed in a manner that favors migration towards the T-cell zone. In addition, it has not yet been explained how this model fits with the T-cell entry and exit strategy from the LNs. However, data from these studies indicate that leukocyte migration behavior to a specific chemoattractant may vary depending on other coexisting chemokine fields, their physiological gradient strength, state of the ligand (soluble versus bound), and tissue-matrix components [37-40,57].

### Role of CCR7 in protective immunity

The role of CCR7 and its ligands in leukocyte guidance is most evident during T cell entry into the LN via HEVs and DC entry through the afferent lymph vessels [2,6,58,59]. Immature DCs continuously sample the antigen milieu in the tissue. When they sense danger signals, they exit as CCR7+ mature DCs via the afferent lymphatics to present antigen in the lymphoid tissues [60,61]. In addition, CCL21-coated stromal networks allow antigen-experienced DCs to establish physical contact with T cells [6,58,59]. CCR7 also appears to be crucial for DC migration from other peripheral tissues such as gut and lung to their corresponding draining LNs [29,30]. These steady state DCs have recently been shown to contribute to T-cell homeostasis by producing vascular endothelial growth factor [62], thus they are able to support HEV formation and may facilitate T-cell entry into the LNs [62]. Such steady state DCs further influence T-cell populations within the LN by increasing CCL21 expression on fibroblastic reticular cells and by their ability to bind free CCL21 [62]. This binding ability is CCR7-independent, but will result in the retention of CCL21 in LNs [62]. Whether or not signaling or behavior of antigen-dependent interactions between DCs and T cells is affected when the DC is also ‘presenting chemokine’ remains to be investigated.

CCR7-deficient or *plt/plt* mice (lacking CCL19 and CCL21-Ser within secondary lymphoid organs) display gross alterations in the micro-architecture of spleen and thymus, and their DCs and T cells fail to home to the spleen and LN T-cell zones [2,59,63]. Consequently, CCR7-deficient mice display delayed and impaired adaptive immune responses, especially in the absence of large amounts of antigen [2,35]. Because antigen presentation at ectopic sites might also activate T cells and produce immunity, the requirement of CCR7 for protective immune responses may vary depending on the pathogen. Although CCR7-deficient mice displayed reduced T-cell priming after infection with lymphocytic choriomeningitis virus or *Listeria monocytogenes*, mice were able to generate sufficient immune responses [64,65]. On the single cell level, CCR7^−/−^ CD8+ T cells gained normal effector function, and antiviral protection was complete but delayed in CCR7-deficient mice [64]. Unlike CCR7^−/−^ mice, *plt/plt* mice were able to mount unimpaired antiviral CTL responses, probably due to the expression of another isoform of CCL21 (CCL21-leu) in lymph vessels and peripheral tissues [66]. However, during the acute phase of infection with the protozoan parasite, *Toxoplasma gondii,* CCR7 appeared to be an absolute requirement for the generation of protective immune responses, despite the systemic nature of this infection and the presence of abundant antigen [43]. This was associated not only with delayed T-cell responses including decreased interferon-γ production, but also a significant defect in the recruitment of inflammatory macrophages [43]. This points to either a direct or indirect role for CCR7 interactions in the recruitment of cells from the bone marrow.

Circulation of T cells between peripheral tissues and lymphoid organs is essential for immune surveillance and host defense of non-lymphoid sites. Upon stimulation by their cognate antigen, naive T cells become activated and change their chemokine receptor expression profile to migrate out of the LN via the efferent lymph [67]. Therefore, inflammation results in a large influx of lymphocytes into peripheral tissues from the bloodstream. Like few other chemokine receptors, CCR7 has been suggested to be a key regulator of lymphocyte migration from blood to specific peripheral tissues [68,69]. Upregulation of CCR7 and/or its ligands has been previously shown in inflamed tissues such as liver, lung, kidney, and muscle [26,70-72] contributing to lymphocyte recruitment and the generation of lymphoid-like structures [25,26].

In a number of studies, CCR7 deficiency led to abnormal lymphocyte accumulation in peripheral tissues such as skin and lung, in which CCR7 is apparently not controlling the entry of activated lymphocytes [24,27]. These observations led to the idea that lymphocytes may also need CCR7 to egress from the body cavities and extralymphoid tissue compartments via the afferent lymph. CCR7 deficiency led to massive accumulation of both CD4+ and CD8+ lymphocytes in the pleural and peritoneal cavities [28]. CCR7 expression is also required for T-cell migration from skin, although CD4+ and CD8+ lymphocytes show differential requirements [27]. CCR7 determines the tissue exit of antigen-specific T cells, and also guides their entry into the mediastinal LN via the afferent lymph of asthmatic lung tissue [24]. DCs residing in tissue surfaces exposed to the external environment such as those in the gut, airway, and skin also use CCR7 to migrate out of the tissues towards peripheral LNs [3,29,30]. Recently, two-photon microscopy used to monitor intralymphatic microinjection demonstrated that CCR7 is dispensable for the parenchymal entry of lymph-derived T cells [73]. However, CCR7 is absolutely required for the directional migration of both DCs and T cells into the T-cell zone in LNs [73]. To summarize, CCR7 orchestrates leukocyte navigation between lymphoid organs and inflamed sites in order to ensure an efficient adaptive immune response.

### CCR7 in central nervous system-specific immune responses

CNS immunity requires the production of appropriate immune responses that are robust enough to control pathogens but at the same time contained to prevent damage in an area in which inflammation is physically restrained by the skull and is dense with sensitive neurons not normally replaced in adult life [74-77]. For decades, the CNS has been considered as an ‘immune-specialized site’ with unique strategies to control the influx of peripheral leukocytes in the brain. Several studies have suggested CCR7 as a potent chemokine signal to control CNS entry and migration of lymphocytes in both healthy and diseased states [42,78-80].

During homeostasis, peripheral immune cells continuously survey for ongoing infection or tissue damage in the brain; however, their migration is restricted. The major route for immune-cell entry and antigen sampling is via the choroid plexus and the meningeal veins into the subarachnoid space [77]. The secretory epithelium of the choroid plexus produces cerebrospinal fluid (CSF) that circulates through the ventricles of the brain. Constitutive expression of CCL19 has been found in the CSF [79], and therefore it is proposed that, after integrin-dependent adhesion, CCR7 signaling may mediate further activation and slowing of lymphocytes on the endothelium to facilitate transendothelial migration [81].

Infection in the periphery leads to the development of a stable, antigen-independent CCR7+ central memory T-cell population with the potential to modulate the quality of T-cell responses after secondary antigen encounter [82,83]. Recent studies on the cellular composition of CSF indicate that most lymphocytes in the CSF express a central memory phenotype with high levels of CCR7 and L-selectin [84,85]. These data, along with evidence of the presence of CCL19 in the CSF of healthy individuals suggest that perhaps as part of routine surveillance, CCR7–CCL19 interactions regulate the entry of central memory T cells into the subarachnoid space [79]. When these cells recognize their cognate antigen, they switch to an effector/activated phenotype, and hence gain access to the CNS parenchyma [86].

In response to inflammatory insults, activated CNS-resident cells induce the expression of adhesion molecules and chemokines that allow circulating leukocytes to bind and extravasate from the cerebral vasculature into the CNS parenchyma [77,87,88]. In experimental autoimmune encephalomyelitis (EAE), an animal model of multiple sclerosis (MS), encephalitogenic T cells extravasate from post-capillary CNS venules [78,81]. Functional expression of CCL19 and CCL21 has been reported in these inflamed CNS venules [78,89]. Blocking of CCR7 signaling reduced binding of T cells to inflamed venules of EAE brain sections [78,81]. In addition, during EAE, CCR7+ cells accumulated in perivascular cuffs and meningeal infiltrates of the brain [89], a finding corroborated further in patients with MS in whom CSF has been found to be enriched with CCR7+ T cells and DCs. By contrast, T cells in MS lesions do not express CCR7, an indication that downregulation of CCR7 after blood–brain barrier transmigration may occur [86]. Similarly, CCL19 transcripts have been found to be upregulated in active and inactive MS specimens [79]. In patients with relapsing-remitting or secondary progressive MS, CCL19 transcript levels correlated with intrathecal IgG production [79]. These data suggest that CCL19 may be involved in B-cell trafficking and expansion in the inflamed CNS. Thus, CCR7 is a potential candidate along with other current B-cell-selective therapeutic approaches to reduce humoral responses in patients with MS [90,91].

Independent of its role in T cell migration, CCR7 signaling has been implicated in the differentiation of T cells [92]. During allergic rhinitis, the absence of CCR7 ligands results in aberrant T helper (Th)2 responses caused by a reduction of Tregs in the cervical LNs (cLN) [93]. By contrast, CCR7 signaling has been found to stimulate DCs to produce IL-23 and IL-12 and to generate Th17 and Th1 cells [92]. Thus, deficiency of CCR7 ligands was found to be protective against development of EAE caused by a defect in IL-23 dependent induction of Th17 cells [92]. Therefore the role of CCR7 in polarizing the immune response is context-specific and may act more as a co-stimulatory signal, as previously reported for CCR5 and CXCR4 [94].

Expression of CCL21 alone is sufficient to recruit lymphocytes and trigger the formation of ectopic lymphoid structures in non-immune-privileged sites [95]. Thus, CCR7 ligands have been thought to contribute to the development of follicle-like structures seen in the meningeal sites of patients with MS [96]. However, transgenic and bioavailable expression of CCL21 in the CNS is not in itself sufficient to promote lymphocyte recruitment or lymphoid neogenesis in the healthy CNS [80,97]. Despite massive lymphocyte infiltration during chronic inflammation, transgenic expression of CCL21 did not form lymphoid-like structures in the CNS parenchyma [80]. Thus, there is no essential link between CCL19/CCL21 expression and ectopic lymphoid tissue formation in the CNS or during MS.

In recent studies, CCR7 has emerged as a potent regulator of neuroimmune crosstalk during inflammation in the brain. CNS-resident glial cells induce CCR7 ligands in response to inflammatory insults in the brain [98]. Although few reports have suggested CCR7 expression by glial cells [98,99], the predominant cell types expressing CCR7 in the CNS are infiltrating CCR7^high^ lymphocytes and dendritic cells [85,86]. In models of infection-induced inflammation, CCR7 and its ligands have been clearly shown to play a protective role. Administration of CCL21/CCL19 restored T cell dysfunction seen in lymphotoxin-α^/-^ mice during viral infection, and increased their resistance to encephalitis [100]. In addition, profound changes in the expression of CCR7 and its ligands have been detected in the CNS during the chronic phase of *T. gondii* infection [42,43]. CCL21 induction appeared as fibrous strands associated with migrating T cells [42]. This may suggest that upregulation of CCL21 can act as a migratory network within the brain parenchyma (Figure 2a) [42]. Indeed, CD4+ T-cell migration in the chronically infected brain parenchyma is CCL21-dependent, and infection is not controlled in the absence of CCL21 [80]. Thus, CCL21 upregulation has been suggested to contribute to CNS protective immunity via: 1) homeostatic priming of CD4 + T lymphocytes outside the CNS and 2) facilitation of CD4 + T cell migration into parenchymal sites within the CNS after pathogenic insults [80].

CCL21 has also been implicated in glial activation in non-infectious CNS insults [50,101]. However, several findings in these models contrast with what has been established from models of autoimmune responses or situations in which ongoing inflammation is present due to CNS infection. Firstly, CNS infection or autoimmune models report expression of CCR7 ligands on glial cells, brain endothelial cells or choroid plexus epithelium [79,86]. By contrast, in both cerebrovascular ischemia and glutamate-mediated damage, neurons are the source of CCL21 production [101,102]. Supporting this finding, endangered cortical and hippocampal neurons have been shown to release CCL21 *in vitro*[101]. However, no CCL21 mRNA was detected from cultured astrocytes or microglia [102,103]. Secondly, although neuronal release of CCL21 leads to microglial activation, this is not via CCR7 but rather another low-affinity receptor, CXCR3 [50,102,104]. In other models of neurological damage such as during peripheral nerve or spinal cord injury, CCL21 is released by damaged neurons [105,106]. This stress-induced CCL21 can be transported as secretory vesicles along neuronal processes and be released at distant sites from the source of injury [101]. This phenomenon indicates a direct mechanism by which damaged neurons communicate and remotely activate microglia or other CCL21-responsive cells (Figure 2b) [105,106]. Thus, CCL21-mediated signaling contributes during neuronal–immune-glial cell interactions, and is required to mount appropriate immune responses during both infectious and non-infectious insults.

## Conclusion

CCR7 has both soluble and immobilized signaling components that are able to exist in a gradient or uniform manner. Its pleiotropic roles in guiding leukocyte migration make it a powerful chemokine system for protective immunity during homeostasis and disease states. The CNS has been considered as an immune-privileged site because of the presence of the blood–brain barrier and the absence of a draining lymphatic system limiting the sampling of antigens present in the brain. However, more recent data have shown that the CNS is not immune isolated; rather, it has intrinsic and extrinsic components that modulate immune responses to maximize their efficiency. Previously known for its crucial role as a homeostatic chemokine in lymphoid organs, CCR7–CCL21 interactions are key to regulating immune responses at and within the CNS tissue. CCR7- dependent migration of leukocytes may play a role in pathogen dissemination in specialized peripheral tissues such as the brain. Thus, careful dissection of the roles of CCR7 signaling in different models of CNS immune responses will be useful in understanding leukocyte trafficking during autoimmune responses or infection.

## Abbreviations

3D, Three-dimensional; CCR, C-C chemokine receptor; CNS, Central nervous system; CSF, Cerebrospinal fluid; DC, Dendritic cells; EAE, Experimental autoimmune encephalomyelitis; GRK, G-protein-coupled receptor kinase; HEV, High endothelial vessel; LN, lymph node; MS, Multiple sclerosis; Th, T helper; Treg, Regulatory T cell.

## Competing interests

All authors declare that there are no competing interests.

## Author’s contributions

SN and EHW wrote the manuscript and designed the figures. All authors have read and approved the final version of the manuscript.
